# The *Lancet* on Narcotics

**Published:** 1884-10

**Authors:** 


### THE *LANCET* ON NARCOTICS.

"Again we have to record with deep regret a sad proof that those who give or take Chloral, or Bromide of Potassium, for sleeplessness are guilty of a deplorable error and do a grievous wrong. The narcotics which poison sleep also deprave the higher nervous centres, enfeeble the controlling power of the will, and leave the mind a prey to the depressing influence of a conscious loss of self-respect and self-confidence. The cultured mind feels the ignominy of this intellectual and moral depreciation with great acuteness, and in the end succumbs to the sense of powerlessness to recover self-control and do right. The deprivation wrought is purely physical. The baneful influence of the lethal drug is, so to say, organic. The essential elements of the nerve tissue are blighted by the stupefying poison, as by Alcohol in habitual drunkenness. In short, the recourse to Chloral and Bromide is precisely the same thing as a recourse to Alcohol. The man or woman who is sent to 'sleep'—the mocking semblance of physiological rest [Will not this apply to all soporifics?]—by a dose of either of these narcotizers is simply *intoxicated.* No wonder habitual drunkenness of this class first impairs and then destroys the vitality of the mind organ, and places the subject of a miserable artifice at the mercy of his emotional nature, and makes him the creature of his passions. When will the public awake to the recognition of facts with regard to the use of these most pernicious stupefacients? Persistence in recourse to them has no better excuse than unwillingness to take the trouble to search out the cause of the 'wakefulness' which prevents natural sleep."

